# Identification of Potential Genes Responsible for Thermotolerance in Wheat under High Temperature Stress

**DOI:** 10.3390/genes10020174

**Published:** 2019-02-25

**Authors:** Peipei Su, Cai Jiang, Hao Qin, Rui Hu, Jialu Feng, Junli Chang, Guangxiao Yang, Guangyuan He

**Affiliations:** 1 The Genetic Engineering International Cooperation Base of Chinese Ministry of Science and Technology, The Key Laboratory of Molecular Biophysics of Chinese Ministry of Education, College of Life Science and Technology, Huazhong University of Science & Technology, Wuhan 430074, China; ppsu886@163.com (P.S.); chloe@hust.edu.cn (R.H.); fengjialu523@163.com (J.F.); cjl@hust.edu.cn (J.C.); 2 Wuhan Igenomics Biotech Inc., Wuhan Oversea Scholar Business Park, East Lake High-Tech Development Zone, 73 Guangguchuangye Street, Wuhan 430075, China; jchnsdu@hotmail.com (C.J.); qhao669@163.com (H.Q.)

**Keywords:** wheat, heat stress, transcriptome profiling, differential expressed genes, Gene Ontology, pathway analysis

## Abstract

Wheat, a major worldwide staple food crop, is relatively sensitive to a changing environment, including high temperature. The comprehensive mechanism of heat stress response at the molecular level and exploitation of candidate tolerant genes are far from enough. Using transcriptome data, we analyzed the gene expression profiles of wheat under heat stress. A total of 1705 and 17 commonly differential expressed genes (DEGs) were identified in wheat grain and flag leaf, respectively, through transcriptome analysis. Gene Ontology (GO) and pathway enrichment were also applied to illustrate the functions and metabolic pathways of DEGs involved in thermotolerance of wheat grain and flag leaf. Furthermore, our data suggest that there may be a more complex molecular mechanism or tighter regulatory network in flag leaf than in grain under heat stress over time, as less commonly DEGs, more discrete expression profiles of genes (principle component analysis) and less similar pathway response were observed in flag leaf. In addition, we found that transcriptional regulation of zeatin, brassinosteroid and flavonoid biosynthesis pathways may play an important role in wheat’s heat tolerance. The expression changes of some genes were validated using quantitative real-time polymerase chain reaction and three potential genes involved in the flavonoid biosynthesis process were identified.

## 1. Introduction

Various climatic factors, such as light, water and temperature, all have great influences on crop production. In recent years, with the continuous increase in greenhouse gases, the global temperature has increased continuously. The high temperature, which could shorten the life cycle by reducing the duration of plant development phases, has become an important factor that adversely influences crop cultivation and productivity [1,2]. As one of the most important food crops cultivated worldwide, wheat is no exception. Like other sessile organisms, wheat can also not escape or keep away from high temperature; therefore, it develops its own regulatory network system, including physiological and biochemistry processes to cope with the adverse environment.

Previous reports showed that plants exhibited significant changes in phenotypic or physiological indices when challenged with high temperatures [3], which always causes serious impacts on yields of grain crops, causing major losses in global agricultural production [4,5,6]. It is estimated that each degree rise in temperature will result in a 6% decrease in global wheat production [7]. Hence, to meet the increasing demand for grain yield and ensure the safety of wheat productivity and quality, it is important to dissect the molecular mechanisms underlying heat tolerance and develop heat-resistant cultivars in wheat in the future.

In response to high temperature stress, plants trigger multiple pathways involved in signal transduction, the scavenging of reactive oxygen species (ROS) and the maintenance of cell membrane stability to ameliorate damage and sustain cell homeostasis [8,9]. The identification of related functional genes or proteins that are responsible for heat stress response (HSR) would be valuable to provide effective strategies to enhance thermotolerance and facilitate understanding of the molecular mechanism in heat stress responses [10]. Some HSR genes have been identified and analyzed. For example, a previous report showed that a homolog of filamentous temperature-sensitive H protease (FtsH), *AtFtsH11*, is involved in the thermotolerance of *Arabidopsis* plants via its regulatory functions in maintaining the thermostability of photosystems under elevated temperatures above the optimum [11,12]. Overexpression of *TaFBA1*, a F-box protein gene isolated from wheat, enhanced the heat stress tolerance of transgenic tobacco [13]. Overexpression of the *TaGASR1* gene in *Arabidopsis* and wheat plants enhanced the thermotolerance of transgenic plants [14]. Additionally, ectopic overexpression of *TaWRKY1* and *TaWRKY33* in *Arabidopsis* led to improved thermotolerance to high temperatures [15]. As the various stress responses are a series of complex biological processes or regulatory networks, the study of a single gene is not always enough to elucidate the tolerance of plants [16,17]. Further studies on genome-wide gene regulation and connection among different regulation pathways on stress response are necessary.

Transcriptome sequencing is a useful and powerful tool for discovering differentially expressed genes (DEGs) and analyzing transcriptome changes of genes under different biotic or abiotic stresses [18,19,20]. Among the existing transcriptome analyses, heat shock proteins (HSPs) are the most frequently and quantitatively observed HSR genes under heat stress of plant species [17,21]. Heat shock proteins are proteins with similar functions. The families of HSP genes are complex in plants; apart from their participation in protein quality control, like prevention of aggregation or denaturation and folding or refolding of proteins under an adverse environment including heat stress, members of this family are also involved in normal growth and development in plants [22,23]. In addition, another frequently mentioned and studied family is the genes encoding heat stress transcription factors (HSFs), which are always involved in mediating the expression of HSPs [17]. The molecular mechanism is complex in heat stress response, including signal cascades and transcriptional control, and not only the expression of related HSPs and HSFs [24], but also the increasing production of antioxidants [25,26], osmoprotectants and cross-talks among various hormonal regulations [27].

Although some transcriptome profiling under heat stress has been investigated in wheat [28,29,30], the participation of genes related with signal transduction pathways or metabolites conversion in this process still needs further research. In this study, we analyzed transcriptome sequencing data from a public resource to further understand the molecular mechanism of the thermoresponse of wheat and to find candidate heat-tolerant genes for wheat improvement.

## 2. Materials and methods

### 2.1. Plant Materials and Heat Treatments

Wheat plants (*Triticum aestivum* L. cv. Chinese Spring) were grown in plastic pots in trays in a greenhouse until the grain filling stage, under a 14 h light/8 h dark photoperiod at 24 °C with a photon flux density of 350 μmol m^−2^ s^−1^ irradiance. Plants in each tray were moved randomly every 3–5 days during the growth period before treatment to avoid positional effects in the greenhouse. Subsequently, wheat plants (15 days after anthesis) were transferred to a high-temperature (37 °C) growth chamber for heat treatment. There were three replicas of treatment and each treatment had three biological repeats. Flag leaves and grains experiencing 37 °C heat stress for 0 min, 5 min, 10 min, 30 min, 1 h, and 4 h were harvested, respectively, snap-frozen in liquid nitrogen and stored at −80 °C for further use.

### 2.2. RNA Extraction and Validation of Gene Expression by Real Time-Quantitative Polymerase Chain Reaction

To test the reliability of RNA sequencing (RNA-seq) data and exploit the key genes responsive to heat stress in wheat, eight upregulated genes were selected for a quantitative real-time polymerase chain reaction (RT-qPCR). Gene names and specific primers designed are listed in Supplementary File 3: Table S1. The total RNA was isolated using Plant Total RNA Extraction kit (Zomanbio, Beijing, China). After removal of genomic DNA with gDNA buffer (gDNase), the single-stranded cDNA was synthesized using 2 μg RNA, FQ-RT primer mix, and RT enzyme mix with the First-strand cDNA Synthesis kit (Tiangen, Beijing, China). The RT-qPCR analysis was performed on a CFX Connect^TM^ Optics Module Real-Time System (Bio-Rad, Hercules, CA, USA) using SYBR Green PCR kits (Vazyme, Nanjing, China) according to the manufacturer’s instructions. *TaActin* (accession no. AB181991.1) was used as the control gene. The RT-qPCR conditions were 95 °C for 15 min followed by 40 cycles of 95 °C for 10 s, 58–60 °C for 15 s, and 72 °C for 30 s. The relative gene expression levels for RT-qPCR data were calculated using the 2^−ΔΔCt^ method [31]. All reactions were conducted in triplicate.

### 2.3. Gene Expression Analysis

We used publicly available data (NCBI Accession: PRJNA427246) for the heat stress-responsive transcriptomes in wheat (*Triticum aestivum* L. cv. Chinese Spring). We collected data on 18 grain and 18 flag leaf samples that were exposed to a high-temperature environment for different periods of time (Supplementary File 3, Table S3).

First, the raw sequencing files from ENA were downloaded. Quality Control (QC) for the RNA-seq reads was checked by FastQC. The reads in the FASTQ files were aligned to the wheat genome (IWGSC) with Spliced Transcripts Alignment to a Reference (STAR) [32]. Then featureCounts was applied to assign reads to genes, and R packages (edgeR) were applied to the transcriptional profiles and tested for differential expression among five different heat exposed grain/flag leaf samples (5 min, 10 min, 30 min, 1 h, and 4 h) with normal grain/flag leaf samples. *p*-values were FDR (False Discovery Rate) adjusted for multiple-testing by computing q-values. Then the significant DEGs (*p* < 0.01 and |log_2_FC| ≥ 2) involved in heat stress response were screened out for further analysis.

### 2.4. Gene Ontology Term and Pathway Enrichment Analyses

The gene ontology (GO) functional enrichment analysis was conducted using the Biomart Database to study the functions of DEGs, and Kyoto Encyclopedia of Genes and Genomes (KEGG) pathway annotation of DEGs was completed using BLASTP to align to KEGG database with a cutoff e-value of 10^−5^ [33,34]. GO enrichment analysis provides all terms that significantly enriched in DEGs and filters out the DEGs corresponding to biological functions. We first mapped all DEGs to GO terms in the database [35,36], then hypergeometric tests were used to find significantly enriched GO terms in DEGs. Corrected *p*-value ≤ 0.01 was taken as a threshold using Bonferroni correction. Calculating the formula for KEGG analysis was the same as that in GO enrichment analysis, which was used to identify significantly enriched pathways of metabolism or signal transduction in DEGs compared with the whole genome background.

## 3. Results

### 3.1. Data Analysis and Identification of Differentially Expressed Genes

**Figure 1:**
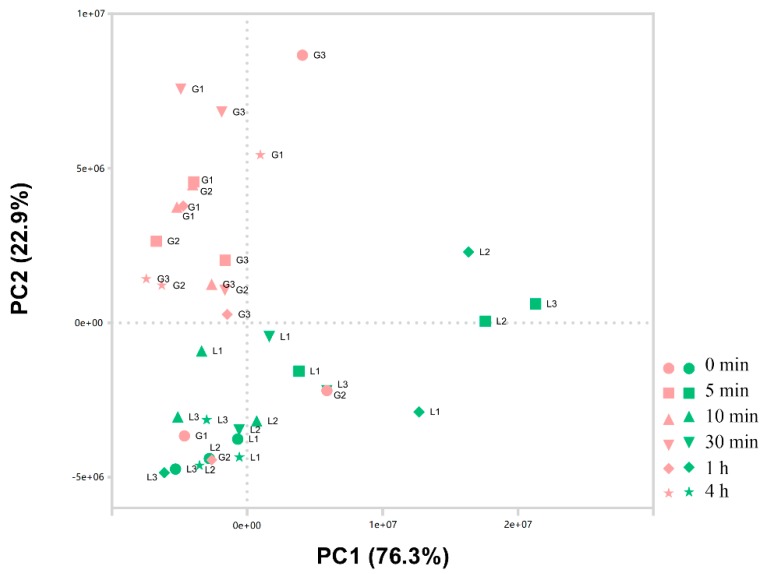
Principal component analysis (PCA) plots of 36 samples from grain and flag leaf groups. G, grain; L, Flag Leaf; L1, 2, 3: Replicas 1, 2, 3. G1, 2, 3: Replicas 1, 2, 3. Triplicate samples from tissues of grain and flag leaf under heat stress (37 °C) for different treatment times (0 min, 5 min, 10 min, 30 min, 1 h, and 4 h) were analyzed.

RNA-seq data of 36 samples from wheat grain and flag leaf, containing three parallel experiments exposed to high temperature (37 °C) for 0 min, 5 min, 10 min, 30 min, 1 h, and 4 h were analyzed. A principal component analysis (PCA) plot showed the gene expression profiles from grain and flag leaf samples located in different regions, which explained the particularly large differences between gene expressions in response to heat stress in grains and leaves (Figure 1). Interestingly, the expression profile from flag leaf was obviously more discrete than that of grain, indicating that the molecular mechanism of flag leaf under heat stress with time may be more complex than that of grain.

**Figure 2:**
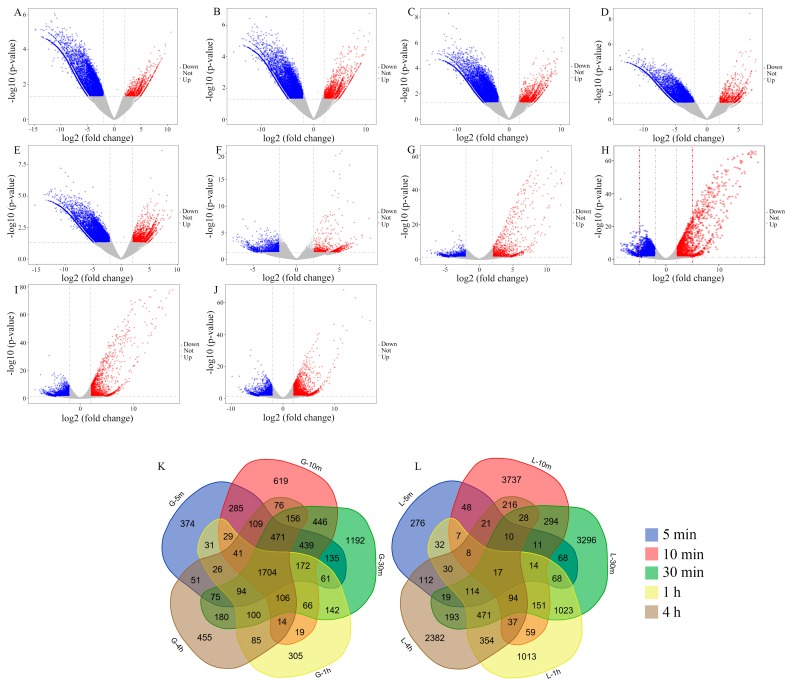
Analysis of differentially expressed genes (DEGs) in wheat grain and flag leaf. Volcano plots represented the differential expressed genes from samples under different times (5 min, 10 min, 30 min, 1 h, and 4 h) of heat treatment in wheat grain (**A**–**E**) and flag leaf (**F**–**J**). Blue and red dots indicate downregulated genes and upregulated genes, respectively; grey dots represent genes that did not display the differences between the heat-treated and normal control samples. Venn diagrams showing heat-regulated genes across five comparisons (5 min/0 min, 10 min/0 min, 30 min/0 min, 1 h/0 min, and 4 h/0 min) in grain (**K**) and flag leaf (**L**) after heat stress treatment. The different colors represent different treatment times under heat stress. G-5 m, 10 m, 30 m, 1 h, and 4 h indicate the differential expressed genes in grain for 5 min, 10 min, 30 min, 1 h, or 4 h treatment.

To further dissect the gene expression profiles under heat treatment with different time durations and between grain and flag leaf, differential expression was assessed from the deposited data. Volcano plots were used to visualize how many transcripts were significantly regulated during heat treatment for different time durations (Figure 2A–J). The middle of each volcano has two lines at which the fold change is +4 or −4, while both sides of the lines indicate downregulation (negative values) and upregulation (positive values), respectively. The significantly up- and downregulated DEGs are represented by red and blue dots correspondingly with criteria of |log_2_FC| ≥ 2 and *p*-values (*p* < 0.01). The results show that the number of upregulated genes was more than downregulated genes in flag leaf after 30 min heat of treatment (Figure 2H). In contrast, downregulated genes were more than the upregulated genes in grain under 30 min of heat treatment (Figure 2C). To further analyze the expression changes under high temperature with different time durations, the overlapped genes of DEGs through five treatment time points were identified. As the Venn diagram shows, 1705 DEGs and 17 DEGs were obtained in grain and flag leaf, respectively (Figure 2K,L). To further analyze the expression pattern changes of DEGs over time, each 10 grouping classes of genes from grain and flag leaf into expression trends over time were also established and the genes from each class were functionally enriched (Supplementary File 3, Figure S1). For example, cluster 1 is a group of genes that downregulated gradually over time; cluster 6 is a group of genes whose expression level was gradually upregulated until a peak 1 h after treatment and then decreased slightly at 4 h of treatment.

### 3.2. Gene Ontology Classification of Differentially Expressed Genes

**Figure 3:**
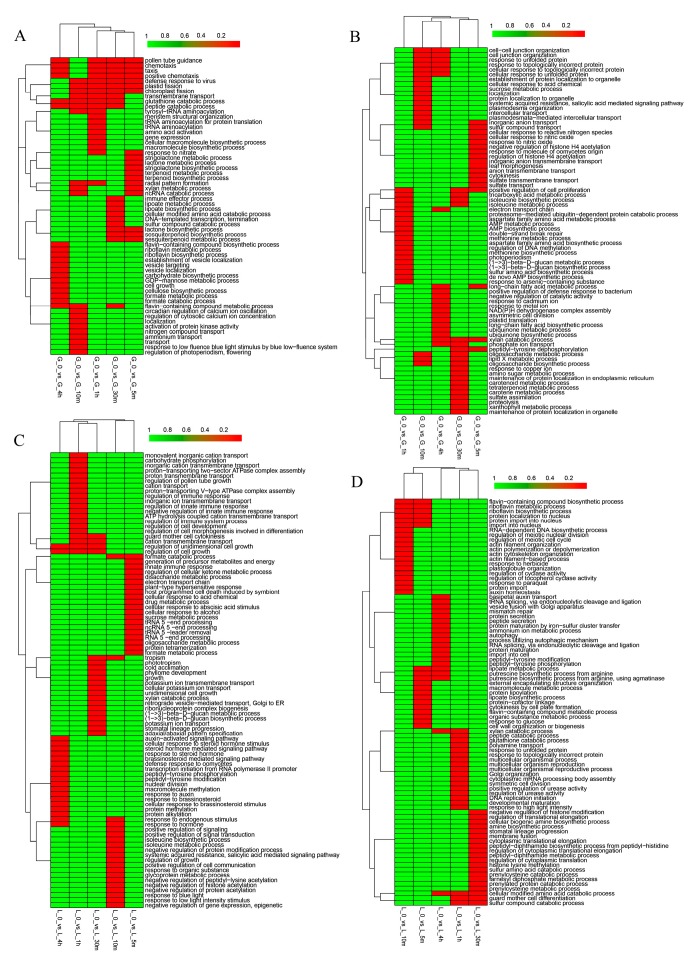
Gene ontology (GO) analysis of the differentially expressed genes for biological process in wheat grain or flag leaf. (**A**) Downregulated genes in wheat grain; (**B**) upregulated genes in grain; (**C**) upregulated genes in flag leaf; and (**D**) downregulated genes in flag leaf.

In order to identify specific molecular factors for the superior heat tolerance in wheat grain and flag leaf, up- and downregulated DEGs caused by heat stress were analyzed by GO term enrichment analysis through different durations of heat treatment. The results show that proteins encoded by upregulated genes were significantly assigned to 18 biological processes (BPs) in the initial response of 5 min in wheat grain, including cellular response to reactive nitrogen species, inorganic anion transmembrane transport, the long-chain fatty acid metabolic process, the xylan catabolic process, and peptidyl-tyrosine dephosphorylation (Figure 3B). At the time point of 4 h of heat treatment, 10 terms of biological processes were specifically enriched, including positive regulation of the defense response to bacterium, negative regulation of catalytic activity, response to cadmium ion, response to metal ion, and NAD(P)H dehydrogenase complex assembly (Figure 3B). In flag leaf, proteins encoded by upregulated DEGs were enriched in 20 biological processes in response to 5-min heat treatment, including innate immune response, electron transport chain, plant-type hypersensitive response, cellular response to acid chemical, and cellular response to abscisic acid stimulus (Figure 3C). In the 4-h heat treatment, proteins encoded by upregulated DEGs were enriched in 20 terms, for example, auxin-activated signaling pathway, brassinosteroid-mediated signaling pathway, transcription initiation from RNA polymerase II promoter, peptidyl-tyrosine phosphorylation, macromolecule methylation, and response to endogenous stimulus (Figure 3C). For the upregulated genes, several functional categories were commonly enriched in grain and flag leaf, including cellular response to acid chemical, sucrose metabolic process, systematic acquired resistance, salicylic acid-mediated signaling pathway, the isoleucine biosynthetic process, the electron transport chain, the xylan catabolic process, and the oligosaccharide metabolic process (Figure 3B,C).

Similarly, we performed a GO classification of genes downregulated by heat treatment for different times. As shown in Figure 3A, the biological functions related to downregulated genes in wheat grain in response to heat stress after as little as 5 min of treatment are the strigolactone metabolic process, lactone metabolic process, strigolactone biosynthetic process, terpenoid metabolic process, terpenoid biosynthetic process, and so on. In response to prolonged thermotolerance (4 h treatment), for example, the flavin-containing compound biosynthetic process, the establishment of vesicle localization, the carbohydrate biosynthetic process, the GDP-mannose metabolic process, and the cellulose biosynthetic process were specifically enriched (Figure 3A). In flag leaf, functional terms were greatly enriched after 5 min of treatment, including the macromolecule metabolic process, protein lipoylation, cytokinesis by cell plate formation, organic substance metabolic process, response to glucose, and cell wall organization or biogenesis (Figure 3D). After 4 h of treatment, the functional categories specifically enriched included basipetal auxin transport, mismatch repair, protein secretion, autophagy, and peptidyl-tyrosine modification (Figure 3D). Detailed data for GO analysis are also given in Supplementary File 1, including the biological process, cellular component and molecular function categories.

### 3.3. Pathway Analysis of Differentially Expressed Genes

**Figure 4:**
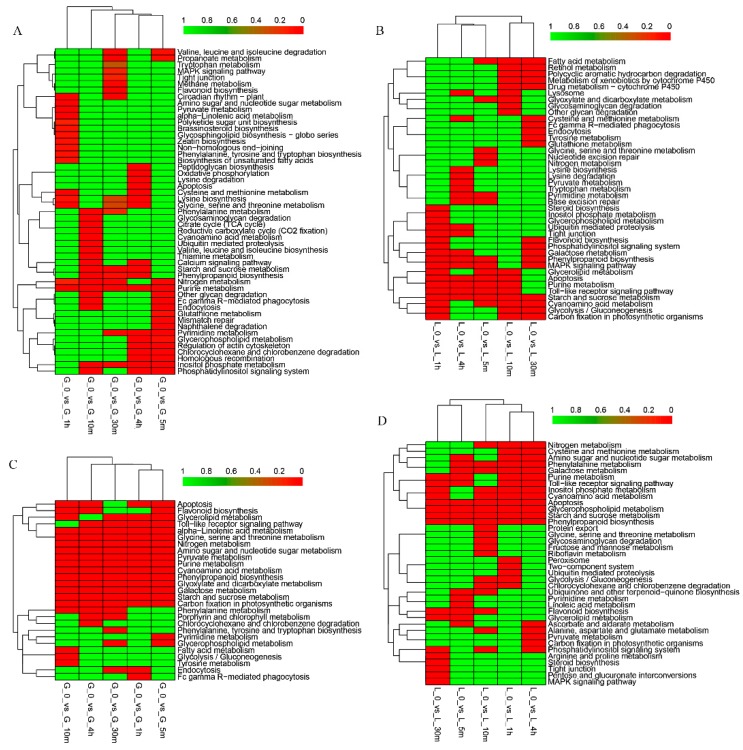
Kyoto Encyclopedia of Genes and Genomes (KEGG) pathway enrichment analysis based on the DEGs. Pathway enrichment analysis based on the differentially upregulated genes (**A**) and downregulated genes (**C**) in wheat grain. Pathway enrichment analysis based on the differentially upregulated genes (**B**) and downregulated genes (**D**) in flag leaf.

To further characterize the biological functions and determine the pathway involvement of these DEGs in heat resistance, KEGG pathway analysis was performed to identify the potential target genes [37]. The upregulated genes in wheat grain have been identified to be involved in alpha−linolenic acid metabolism, brassinosteroid biosynthesis, zeatin biosynthesis, biosynthesis of unsaturated fatty acids, glycerophospholipid metabolism, and so on (Figure 4A). The downregulated genes in wheat grain have been identified to be involved in pathways including nitrogen metabolism, amino sugar and nucleotide sugar metabolism, phenylpropanoid biosynthesis, starch and sucrose metabolism, and carbon fixation in photosynthetic organisms through five different time points (Figure 4C). However, in flag leaf, the significantly enriched pathways of upregulated genes involved were starch and sucrose metabolism through gradually prolonged time heat treatment; in addition, pathways of fatty acid metabolism, glutathione metabolism, phenylpropanoid biosynthesis, glycerolipid metabolism, and flavonoid biosynthesis were enriched (Figure 4B). The predominant pathways of the downregulated genes were enriched in apoptosis, starch and sucrose metabolism, glycerophospholipid metabolism, and phenylpropanoid biosynthesis through all the time points (Figure 4D). The pathways of DEGs commonly identified in wheat grain showed a similar response to different time treatments. In contrast, most pathways presented a time-dependent response in flag leaf, which further implied that the molecular mechanisms in heat response in flag leaf are more complex. Detailed data for the KEGG pathway are given in Supplementary File 2.

### 3.4. Verification of Candidate Differentially Expressed Genes by Real Time-Quantitative PCR

**Figure 5:**
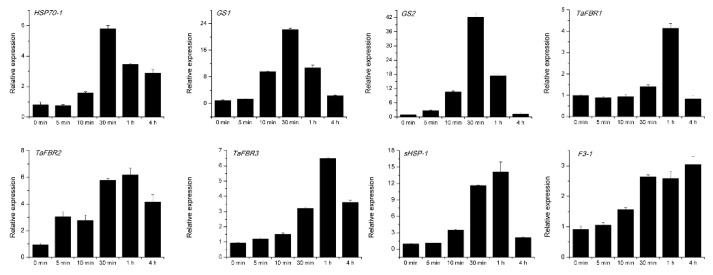
Validation and expression of eight selected genes detected by real time-quantitative PCR (RT-qPCR). *TaFBR1*, *2*, *3*: *Flavonoid biosynthesis related gene1, 2, 3*. *HSP70-1*: the gene encoding heat shock protein 70. *GS1*, *GS2*: the genes encoding glutathione S-transferase. *F3-1*: flavonoid 3′-monooxygenase. Data represents the mean ± SD calculated from three independent replicates. Gene expression levels were normalized to the internal control *TaActin*.

To further test the reliability of RNA-seq data and the expression patterns of the DEGs revealed by transcriptome sequencing, RT-qPCR was performed to examine the expression patterns of eight selected DEGs, including three genes involved in the flavonoid compounds biosynthesis pathways (Figure 5). Real time-quantitative PCR results showed that the expression of the gene encoding heat shock protein 70 (*HSP70-1*) showed a peak expression level after 30 min treatment in grain, and the genes encoding glutathione S-transferase (*GS1*, *GS2*) showed a similar trend with HSP70-1 in flag leaf. Three genes related to flavonoid biosynthesis (*TaFBR1*, *2*, *3*) and the gene encoding small heat shock protein (sHSP-1) all showed a peak expression level at 1 h after treatment and then decreased 4 h after treatment in flag leaf. The transcript of the gene *F3-1*, which encodes flavonoid 3′-monooxygenase, basically kept a gradually increasing expression level over time and peaked 4 h after treatment in flag leaf. These results revealed that the expression trends of these genes were basically in accordance with the transcript abundance changes from RNA-seq data. Existing discrepancies in the expression levels of these genes between RT-qPCR and RNA-seq might have resulted from the errors which existed between repeated experiments or different sensitivity and corresponding algorithms between the two analysis methods.

## 4. Discussion

High temperature is one of the key climatic parameters affecting both plant growth and development, thereby causing extensive loss of crop yield [5,38,39]. Seeds exposed to high temperatures show germination delay and reduced seedling emergence. Heat stress can also limit photosynthesis, increase the photorespiration and rate of transpiration through stomatal regulation, and ultimately lower the biomass of the plants [38,40]. Although the physiological effects of heat stress on crops have been extensively studied, dissection of the heat stress responsive mechanism and identification of key components involved in signal transduction pathways of the heat stress are still limited [41,42]. Plant tissues are all sensitive to high temperature, and the activation of different pathways between tissues of reproductive and vegetable organs may be particularly tissue-specific [42]. 

From volcano plots, more upregulated transcripts in the flag leaf versus those in the developing grain may implicate that the regulation mechanism in the leaves is much tighter and more positive in transcriptional level to sustain the normal physiological process of the leaf to some degree. The number of downregulated genes in grain is significantly larger than that in the flag leaf, which also suggests that responsive genes from seeds may be more sensitive and most of the genes involved in the synthesis of macromolecular compounds for material accumulation during the grain filling stage are inhibited as the grain is the main site for synthesizing and storing starch and protein (Figure 2A–J). In the present study, we also found that the number of DEGs observed in flag leaf responsive to high temperature after 5 min (856) is much lower than that in grain (4098), suggesting that many more genes from the grain are susceptible than in flag leaf. Furthermore, the overlap of DEGs through different durations of heat stress in wheat grain showed that the DEGs overlap more, which possibly indicates that the defense or stress mechanism in grain may not change greatly over time (Figure 2K). In contrast, we showed that the DEGs vary significantly over time under high temperature stress in flag leaf, which suggests that the defense or heat stress mechanism of flag leaf under high temperature stress may change more over time and may be more complex (Figure 2L). 

Grouping classes of genes for expression trends over time and GO analysis of each cluster gene showed that the genes that responded first in the flag leaf at the 5-min time point mainly exhibited a cellular response to ROS, lipid metabolic process, and small molecule biosynthetic process (Clusters 1, 3; Supplementary File 3, Figure S1), followed by genes involved in the nucleoside phosphate catabolic process, histone exchange and stress acclimation that obviously responded at the 30 min time point (cluster 7; Supplementary File 3, Figure S1). In developing grain, more genes responded at the 5-min time point (clusters 1, 3, 4, 6, and 9) and mainly participated in the lipid biosynthetic process, hormone metabolic process, defense response and positive regulation of response to ROS, followed by the genes that responded after 30 min of treatment that were mainly involved in the phosphorylation and fatty acid biosynthetic process (cluster 10; Supplementary File 3, Figure S1). These results suggest that the response of genes to external heat stress is tissue-specific and has a strongly time-dependent manner.

Functional enrichment analysis of the DEGs involved in wheat grain for the BP category showed that, in the case of downregulated genes, they mainly included macromolecule biosynthesis as well as peptide catabolism. This means that the synthesis of macromolecules was inhibited, and the degradation of polypeptide glutathione that protects plants was also downregulated. At the same time, genes related to the gene expression and carbohydrate biosynthetic processes were also downregulated. These results demonstrated that at high temperatures, many metabolism processes of grain were suppressed; downregulation of macromolecule synthesis and carbohydrate biosynthetic processes means that the protein and starch accumulation in grain was inhibited, indicating that the grain filling stage was affected by the high temperature and resulted in a decrease in grain weight and wheat yield. For upregulated DEGs, the BPs of unfolded protein response and (cellular) response to topologically incorrect proteins were enriched, meaning that high temperature induces positive responses to abiotic stresses, such as the production of HSPs [22,23]. Genes related to the functions of cellular response to reactive nitrogen species and (cellular) response to nitric oxide had their expression level increased after 5 min, indicating that active nitrogen and many other reactive oxygen species appear in the early stage of high temperature stress, and plants responded to this stress immediately—as early as the initiation of treatment at 5 min. This suggests crosstalk between HSP and ROS production, showing that, as signaling molecules, the ROS can induce HSPs, which corroborates previous reports [43,44]. Our results suggest the participation of DNA damage as well as energy metabolism reprogramming in the course of heat acclimation [45,46].

In flag leaf, the significantly downregulated BPs were biodegradation processes, such as the peptide catabolic process, the glutathione catabolic process, and the xylan catabolic process, indicating that the metabolism of flag leaves, especially catabolism, decreased after high temperature stress. Upregulated genes have been identified to be involved in pathways including the ion transport channel, such as inorganic cation/ion transmembrane transport, proton transmembrane transport, and regulation of cell growth, indicating that the membrane transport mechanism of flag leaf surface cells changed and ion-dependent signaling pathways may be affected by high temperature stress; the growth of leaf cells changed, too.

For KEGG pathway analysis, similarly, the most significant signal pathways of downregulated DEGs in grain are starch and sucrose metabolism; carbon fixation in photosynthetic organisms; galactose metabolism; glyoxylate and dicarboxylate metabolism; and the downregulated synthesis of starch, sucrose, galactose, and the carbon fixation pathway, which showed the accumulation of assimilate in developing grain was inhibited, and thus, explains the decreased yield under high temperature. Interestingly, the metabolism of flag leaves is disturbed as most significant signal pathways including both up- and downregulated genes have been identified in pathways such as starch and sucrose metabolism, apoptosis, phenylpropanoid biosynthesis, glycerophospholipid and galactose metabolism, glycolysis/gluconeogenesis, flavonoid biosynthesis, and steroid biosynthesis, indicating that the genes involved in these pathways showed both up- and downregulating patterns, which were likely to keep these pathways in balance under heat stress; that is, these biological metabolic pathways undergo dynamic changes under high temperature to cope with heat stress [20]. We also found that linoleic acid metabolism (downregulation), fatty acid metabolism (upregulation) and glycerolipid metabolism (upregulation) were enriched in flag leaf, indicating that the regulation of glycerolipid metabolism at a transcriptional level is important for the heat stress response, which is consistent with the results of previous reports [47,48]. In addition, the common KEGG pathways in grain were obviously more enriched through five time points of heat treatment than those of flag leaf, which further illustrates that the heat response mechanism in flag leaf is more complex.

Previous studies showed that phytohormones, such as ABA, auxin, ethylene, gibberellin, and brassinosteroid, were likely linked to heat stress signaling in different species [17,27,49,50]. In the present study, we found that processes of the secondary metabolism pathway of flavonoid biosynthesis and hormonal pathways of brassinosteroid and zeatin biosynthesis pathways were actively regulated in response to high temperature stress in wheat (Figure 3C and Figure 4A–D). The steroid hormone brassinosteroid can regulate growth and development in plants and has been reported to be responsive to different environmental stresses [42,51]. Recently, Jiang et al. [20] showed that the zeatin and brassinosteroid biosynthesis pathways are likely to play important roles in response to high temperature stress in maize.

Based on the above GO, KEGG pathway analyses and expression profiles of DEGs under different time treatments, our attention was focused on the gene exploitation involved in secondary metabolite biosynthesis pathways. In plants, as some of the representative important secondary metabolites, flavonoid compounds have multiple biological functions and are involved in several aspects of plant development and defense, including pollinator attraction and seed dispersal, ultraviolet filtration, symbiotic nitrogen fixation, and antimicrobial effects [52,53,54]. In addition, flavonoids play an important role in scavenging ROS to enhance stress tolerance, and evidence of flavonoids as antioxidants is accumulating gradually [55,56,57]. Also, high temperature-induced ROS accumulation can be reduced by flavonols to control pollen tube growth and integrity [58]. Furthermore, they are also important as pharmaceutical and nutritional compounds that are beneficial for human health [59].

In the present study, three genes, *TaFBR1*, *TaFBR2*, and *TaFBR3* were identified, which may be very important and have large potential for thermotolerance improvement of wheat. The three genes were screened out based on the *Arabidopsis* genes *C4H (Cinnamate-4-hydroxylase)*, *FLS1 (flavonol synthase*) and *TT5* *(chalcone isomerase*), involved in the flavonoid biosynthesis pathway. Gene functions, especially in resisting adverse environmental conditions involved in the flavonoid biosynthesis, have been frequently studied. For example, a previous report showed that *TaFSL1* enhanced the salinity tolerance of transgenic *Arabidopsis* by primary root elongation compared with the control plants [60,61]. As various stresses often occurred in a combined or interconnected way [62,63], *TaFSL1* was very likely to be responsive to heat stress; this requires further study and validation. Cinnamate 4-hydroxylase belongs to the cytochrome P450 family, a superfamily that is well known for its roles in the degradation of environmental toxins and mutagens, and plays an important role in plant chemical defense, hormone synthesis and decomposition, and secondary metabolism [64]. In *Arabidopsis*, *TT4/CHS* and *TT5/CHI* were the first two enzymes of flavonoid biosynthesis and *Arabidopsis* mutants for *tt4*; *tt5* were reported to be impaired in the protection against oxidative stress [65,66]. In addition, it is known that the flavonoid biosynthesis pathway is regulated by many transcription factors such as the MYB, bHLH, WD40, and WRKY proteins, through sequence-specific DNA binding or protein‒protein interactions [67,68,69]. MYB is particularly important in regulating flavonoid biosynthesis in response to abiotic stresses. Overexpression of *LeAN2* (*R2R3-MYB*) induces the accumulation of anthocyanin, enhanced tolerance to chilling and oxidative stress of transgenic tobacco, and improved thermotolerance of transgenic tomatoes [70,71]. *AtMYB12* was identified as a positive regulator of flavonoid biosynthesis by upregulating the expression of *CHS* and *FLS* genes, while overexpression of *AtMYB12* significantly increased the flavonoid content and enhanced the salt and drought tolerance of transgenic *Arabidopsis* [72,73]. In addition, MYB genes from wheat were proven to be responsive to high temperature and can improve the heat and drought tolerance in transgenic *Arabidopsis* [74]. *GS1* and *GS2*, encoding glutathione-S-transferases, were observed to be responsive to high temperature in our analysis; glutathione-S-transferase is the key antioxidant enzyme and overexpression of glutathione S-transferase/glutathione peroxidase enhanced tolerance to various stresses, including thermotolerance, by providing protection from stress-induced oxidative-damage in transgenic tobacco seedlings [75]. HSPs, including the sHSPs, were always actively regulated in the plant under high temperature environment; the *HSP70-1*, functionally predicted as a *grpE* gene, is a cofactor of HSP70. Overexpression of a *HSP70* gene from *Brassica campestris* confers enhanced tolerance to heat stress in tobacco by removing excess ROS [76]. The over-expressed sHSP gene, *TaHSP26* from wheat imparts thermotolerance in transgenic plants of *Arabidopsis* [77], overexpression of *OsHSP18.6*, enhanced the tolerance to heat stress in rice plants by depressing the damage caused by ROS through participating in the scavenging process of ROS [78]. *F3-1*, encoding a flavonoid 3′-monooxygenase, was also up-regulated under high temperature in the aforementioned result. Flavonoid 3′-monooxygenase is a class of oxidoreductase and is involved in the biosynthesis of flavonoid compounds. *PnF3H* (encoding a flavanone 3-hydroxylase), isolated from the Antarctic moss *Pohlia nutans*, improves the salt stress tolerance in transgenic *Arabidopsis* [79]. Our RT-qPCR analysis showed that the expression trend of these genes basically agrees with the RNA-seq results, with different degrees of inductions under heat treatment. We speculate that these genes may play important roles in heat resistance and their functions need further exploitation and verification.

In addition, a weighted gene co-expression network analysis (WGCNA) was performed to construct the co-expression module; for example, in flag leaf, the genes from the royal blue module were further analyzed in a correlation study among the modules. It was found that the correlation (0.8) between genes from the royal blue module and the phenotype of high temperature treatment time was significantly high. We also found some key genes from the royal blue module; the top 10 are listed in Supplementary File 3, Table S2. These genes may be important in response to heat treatment and may have potential value as candidate gene resources for heat resistance in wheat.

## 5. Conclusions

Transcriptome sequencing data of wheat developing grain and flag leaf under high temperature treatment of 37 °C were comparatively analyzed in this study. A large amount of heat stress-responsive genes were modulated shortly after high temperature stress, indicating the self-adjustment of wheat in response to heat. The gene responses at transcriptional level under heat stress between different organs of grain and flag leaf differed largely and the regulatory mechanism in flag leaf may be more complex and tighter. In addition, our analyses suggest that the heat shock response in wheat grain may be more heat-sensitive than that of flag leaf; it seems that the latter tissue could be better acclimated to elevated temperatures [42]. We further found that the zeatin, flavonoid, and brassinosteroid biosynthesis pathways, especially the flavonoid biosynthesis pathway, were likely to play important roles in regulating the response of wheat to high temperature stress. A number of candidate genes, especially the three genes involved in the flavonoid biosynthesis process, were identified in this study, but further studies are needed to clarify the roles of these genes in the process of heat resistance. Therefore, our results help to further elucidate the mechanism underlying the heat stress responsive mechanism and provide potential candidate genes for improving wheat heat tolerance.

## Supplementary Materials

The following are available online at https://www.mdpi.com/2073-4425/10/2/174/s1, Supplementary File 1: Functional enrichment analysis data of up- and down regulated genes in wheat grain and flag leaf under different treatment times, including three categories of biological process, cellular component, and molecular function; Supplementary File 2: Pathway enrichment analysis data of up- and downregulated genes in wheat grain and flag leaf under different treatment times; Supplementary File 3: Table S1: Primer sequences used in our RT-qPCR analysis; Table S2: Top 10 key genes from wheat grain and flag leaf identified by WGCNA analysis; Table S3: Plant materials and treatment of RNA-seq samples and our experiment; Figure S1: Expression pattern trends of grouping classes of DEGs over time.
