# Supplementary material for: Identification of Potential Genes Responsible for Thermotolerance in Wheat under High Temperature Stress
Source: Genes (Basel). 2019 Feb 25;10(2):174. doi: 10.3390/genes10020174 (PMC6410297; doi:10.3390/genes10020174)
Supplement: Supplementary file 1 [file genes-10-00174-s001.zip › genes-421952-supplementary-major revision/Supplementary File/Supplementary File 3.pdf]

Figure S1

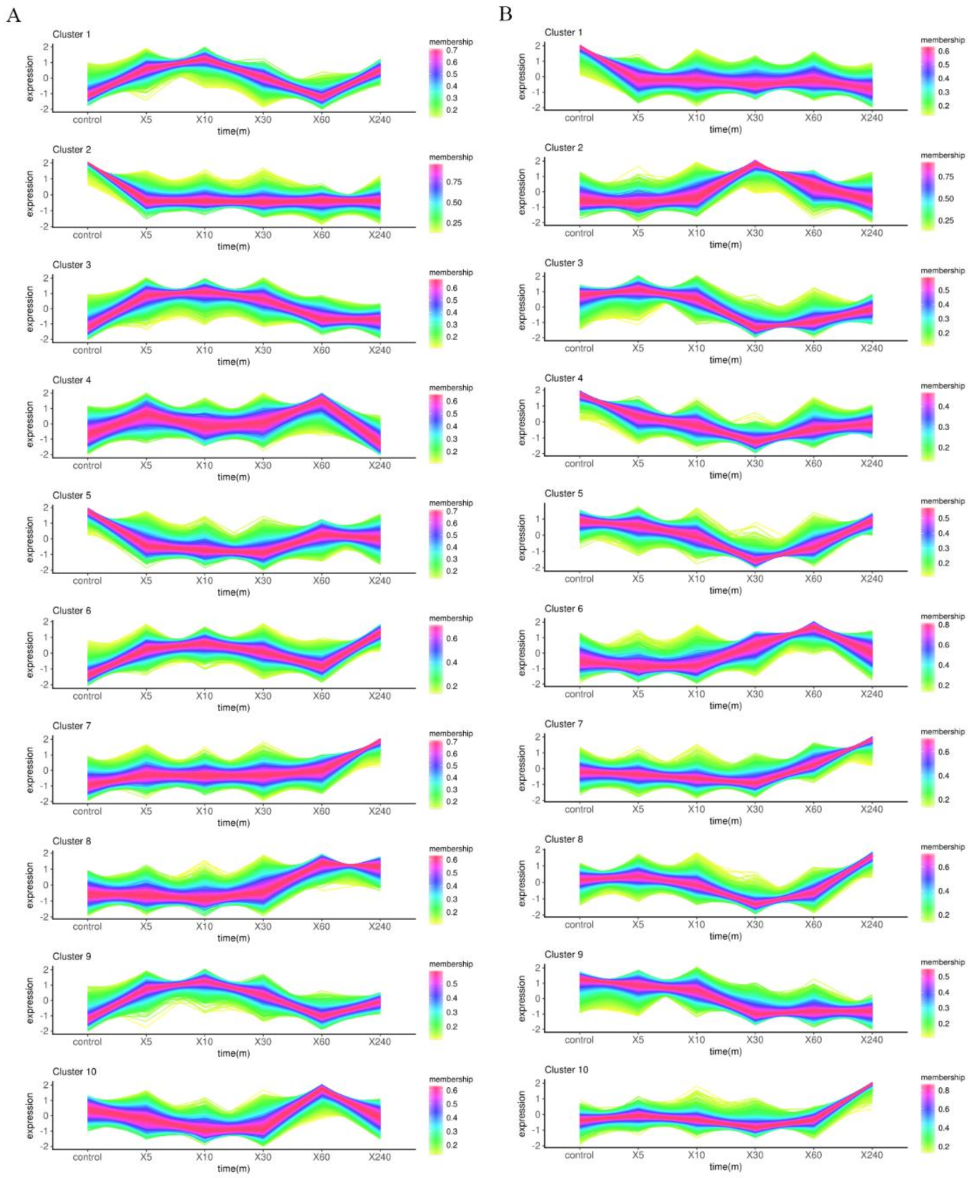

Expression pattern trends of grouping classes of DEGs over time. (A) Ten clusters grouped by differentially expressed genes from developing grain. (B) Ten clusters grouped by differentially expressed genes from flag leaf. The horizontal axes indicated the 5 time points of heat treatment, and the vertical axes indicated the expression trend of gene clusters.

Table S1. List of primer sequence used in this study

Gene	Forward primer	Reverse primer
TaFBR1	GACTTCATCCCCATCCTCCG	CGTTGTCCTCGTTGATCTCCC
TaFBR2	TGATCGACTGCATCTTAGGGTT	TCGGGTCTTGGACACGGA
TaFBR3	CGAAAGATAAATCCCGACACTG	TGATCTGCCTGCCATCAATT
F3-1	CGGGAAGTTCAACATCAGCG	CCTGCTCCAAGCCACCAA
HSP70-1	ATTTCTCCCGTGCCTCCTTG	CGAAGCCGGTCCTTTGGTAT
sHSP-1	CCTCGTCGCGCTGGATTT	ACCGCATCTTCACCTCCTTC
GS1	CACGGGGAAGGCATCTATTG	ACTTGTCGTCCCATTCGAGG
GS2	TGATCGACAAGGGGAGGACT	TCTAAACTCTCAAGCCCCTGC
TaActin	AGTGGAGGTTCTACCATGTTTCCT	CACTGTATTTCCTTTCAGGTGGTG

Table S2. Top 10 key genes from wheat grain and flag leaf respectively identified by WGCNA analysis

Gene (Flag leaf)	Correlation	Gene (Grain)	Correlation
TRIAE_CS42_2BS_TGACv1_152915_AA0499060	0.984445246	TRIAE_CS42_1AL_TGACv1_000771_AA0018790	0.989149303
TRIAE_CS42_3DL_TGACv1_252573_AA0890770	0.979265308	TRIAE_CS42_3AL_TGACv1_194869_AA0641000	0.987944028
TRIAE_CS42_5AL_TGACV1_374280_AA1195930	0.972906211	TRIAE_CS42_3DL_TGACv1_251724_AA0884250	0.981640085
TRIAE_CS42_3DS_TGACv1_271880_AA0910080	0.972262551	TRIAE_CS42_4DL_TGACv1_346552_AA1155850	0.980469815
TRIAE_CS42_5AS_TGACV1_393222_AA1270040	0.971243151	TRIAE_CS42_1BL_TGACv1_033860_AA0142380	0.980002224
TRIAE_CS42_7BL_TGACV1_579404_AA1907000	0.969379356	TRIAE_CS42_2AL_TGACv1_093753_AA0286070	0.979839942
TRIAE_CS42_3DL_TGACv1_249244_AA0842940	0.965803625	TRIAE_CS42_7DL_TGACv1_602597_AA1962040	0.978970112
TRIAE_CS42_5AS_TGACV1_392698_AA1263180	0.965573848	TRIAE_CS42_U_TGACv1_644701_AA2141240	0.978005102
TRIAE_CS42_2BS_TGACv1_149714_AA0497520	0.965331988	TRIAE_CS42_4DL_TGACv1_342934_AA1125640	0.975640157
TRIAE_CS42_5AL_TGACv1_374527_AA1202410	0.964876512	TRIAE_CS42_6DL_TGACv1_526957_AA1696050	0.971661782

Table S3. Plant materials and treatment of our experiment and RNA-seq samples

Samples selected	Ttreatment temperature	Treatment time	Growth stage	Sampling time (stage)	Cultivar	Replicas
Grain	24℃	0	Grain filling stage	15 days after anthesis	*Triticum aestivum* L. cv. Chinese Spring	3
Leaf	24℃	0	Grain filling stage	15 days after anthesis	*Triticum aestivum* L. cv. Chinese Spring	3
Grain	37℃	5 min, 10 min, 30 min, 1 h, 4h	Grain filling stage	15 days after anthesis	*Triticum aestivum* L. cv. Chinese Spring	3
Leaf	37℃	5 min, 10 min, 30 min, 1 h, 4h	Grain filling stage	15 days after anthesis	*Triticum aestivum* L. cv. Chinese Spring	3
